# Motility Profile of Captive-Bred Marmosets Revealed by a Long-Term In-Cage Monitoring System

**DOI:** 10.3389/fnsys.2021.645308

**Published:** 2021-04-15

**Authors:** Masashi Koizumi, Naotake Nogami, Kensuke Owari, Akiyo Kawanobe, Terumi Nakatani, Kazuhiko Seki

**Affiliations:** Department of Neurophysiology, National Institute of Neuroscience, National Center of Neurology and Psychiatry, Tokyo, Japan

**Keywords:** non-human primate, marmoset, home-cage activity, neurodegenerative diseases, passive infrared pyroelectric motion sensor, intermittency of movement, daily activity profile

## Abstract

A quantitative evaluation of motility is crucial for studies employing experimental animals. Here, we describe the development of an in-cage motility monitoring method for new world monkeys using off-the-shelf components, and demonstrate its capability for long-term operation (e.g., a year). Based on this novel system, we characterized the motility of the common marmoset over different time scales (seconds, hours, days, and weeks). Monitoring of seven young animals belonging to two different age groups (sub-adult and young-adult) over a 231-day period revealed: (1) strictly diurnal activity (97.3% of movement during daytime), (2) short-cycle (∼20 s) transition in activity, and (3) bimodal diurnal activity including a “siesta” break. Additionally, while the mean duration of short-cycle activity, net daily activity, and diurnal activity changed over the course of development, 24-h periodicity remained constant. Finally, the method allowed for detection of progressive motility deterioration in a transgenic marmoset. Motility measurement offers a convenient way to characterize developmental and pathological changes in animals, as well as an economical and labor-free means for long-term evaluation in a wide range of basic and translational studies.

## Introduction

To move and explore the surrounding environment is crucial for animals. Motility is vital for maintaining strength and the flexibility of behaviors required to survive under a constantly changing environment. Motility declines following functional deterioration of multiple body parts (Ferrucci et al., 2016; Buchman et al., 2018). Movement disorder often accompanies certain neurologic diseases (Taroni and Didonato, 2004; Nóbrega and De Almeida, 2012), severely limiting an individual’s viability (Barlow et al., 1996; Robinson et al., 2013). Behavioral phenotyping of experimental animal models of human disease aims to quantitatively characterize the motor behavior and its disease-specific deviation from healthy animals (Lin et al., 2001; Pratte et al., 2011; Urbach et al., 2014). Measurement of bodily movement (e.g., locomotion or ambulation) is often included in the established battery of assays used to evaluate an animal’s behavior (Crawley and Paylor, 1997; Rogers et al., 1997). It offers a practical way to compare the level of motility among individuals with different genetic backgrounds (Tang et al., 2002), disease or disease progression (Portal et al., 2013). Motility measurements can reflect various factors, such as changes in the external environment (Casadesus et al., 2001; Barbosa and Mota, 2009) or internal metabolic states (Garland et al., 2011). Traditionally, measurements of this type have been performed though multiple iterated sessions under strictly controlled protocols, i.e., duration and interval of measurement, dimension and condition of measurement field (Wahlsten, 2001), so as to minimize test-irrelevant variation. However, this kind of measurement is highly labor-intensive for both the animal and the experimenter. This is particularly taxing when performing repeated, rigorous tests to evaluate alterations in longitudinal motility, which frequently occur with a very slow time constant (e.g., over months and years) during maturation, aging, and disease progression (Carter et al., 1999; Yhnell et al., 2016). An easy and efficient way to measure the motility of animals through their life span is, therefore, essential to overcome this limitation.

In-cage motility evaluation, whereby the experimenter measures motility exclusively within the home cage, in which each animal spends their normal life, has been applied recently in rodents (Spruijt and Devisser, 2006; Redfern et al., 2017). This method has a number of advantages over traditional iterated measurements. First, it is more labor friendly. Second, it achieves a remarkable gain in long-term consecutive output, enabling the evaluation of time dependency, periodicity, and long-lasting, slowly developing alterations, such as those associated with aging or progressive disease. Third, by introducing automated, continuous measurement without involvement of the experimenter during evaluation, it improves the reproducibility and replicability, which were typically sacrificed in the iterated approach because of uncontrollable fluctuation of environmental factors as well as experimental procedures (Crabbe et al., 1999).

Here, we report an in-cage motility measurement method specifically developed for non-human primates. These species represent superior models of human disease owing to their phylogenetic proximity, as well as structural and functional similarity to humans (Capitanio and Emborg, 2008; Kishi et al., 2014; Phillips et al., 2014; Walker et al., 2017). Specifically, the common marmoset (Callithrix jacchus) has become a valuable primate species in translational studies of human disease (’t Hart et al., 2015; Galvao-Coelho et al., 2017), including those relating to movement disorder (Emborg, 2017). Their small body size makes them easy to handle, saves space, and allows breeding a number of disease models in a single laboratory. Their husbandry is comparable to that of rodents, which is particularly advantageous in comparison with larger primate species such as macaque monkeys. However, so far, behavioral phenotyping of marmosets has been more challenging than for rodent models. There are no established battery tests. The number of animals per study is limited by ethical (Prescott, 2010) and economic reasons. A divergent genetic (Barr et al., 2003) and environmental (Johnson et al., 1996) background leads to different physiological and psychological profiles among individuals (Slipogor et al., 2016; Koski et al., 2017). The common marmoset is generally a stress-sensitive primate species and is readily disturbed by an unusual situation or following separation from mates (Norcross and Newman, 1999; Bassett et al., 2003; Kaplan et al., 2012). Marmosets are arboreal animals (Garber, 1992; Larson, 2018) and thus they prefer to move vertically, not only horizontally. Finally, their longer life span (Abbott et al., 2003; Nishijima et al., 2012) makes frequent measurement for each stage of life very challenging. Consequently, so far, phenotyping marmoset behavior has relied on *ad hoc* methods (Marshall and Ridley, 2003; Verhave et al., 2009; Choudhury and Daadi, 2018) or on mere objective descriptions.

The in-cage motility monitoring system we propose here is intended to overcome these limitations as it is specific to non-human primates. It enables motility to be assessed with minimal stress by performing measurements daily under a familiar environment for both marmosets and human experimenters, it avoids inter-individual differences in habituation to the specific measurement field and equipment, it can easily capture any movement that occurs within the three-dimensional space of the home cage, and could potentially measure the motility continuously throughout an animal’s life span. The aim of this study was to validate in-cage motility measurement on various marmoset age groups (from adolescent to elderly), healthy and disease model, and under different times of diurnal and annual cycles. We developed an off-the-shelf measurement system based on a passive infrared movement detector and data-logger capable of running uninterrupted 24 h a day for months. Using this novel system, we successfully characterized the motility of healthy common marmosets and transgenic models of human disease, on different time scales ranging from second to weeks.

## Methods

### Experimental Set-Up

#### Ethics Statement

This study compared the motility of wild-type marmosets from two different age groups (Experiment 1), and that of a transgenic marmoset model of human ataxia with age-matched control groups (Experiment 2). All procedures and housing conditions were approved by the committee on the ethical issue in animal experiments of the National Center of Neurology and Psychiatry (NCNP) and were carried out in accordance with its guideline for the care and use of primates. The latter complied with the guideline issued by the Japanese Ministry of Education, Culture, Sports, Science and Technology (2006) and the National Institutes of Health guide for the care and use of Laboratory animals (National Research Council (US) Institute for Laboratory Animal Research, 1996).

#### Housing Environment

All marmosets used in this study were housed in a breeding room of the primate research facility at the NCNP. In the breeding room, 54 cages were divided into two series and placed along the walls. This layout facilitated social contact as it allowed marmosets to hear or see each other. The room was maintained at 28°C and 50% humidity by an automatic air conditioning system. Lighting was kept under a constant artificial 12-h dark/12-h light cycle managed by a control timer that regulated the fluorescent lamps on the ceiling. Illuminance near the floor of the room was 450 lux. Water was freely supplied via a feeding bulb attached to the cage. The diet consisted mainly of pellets adjusted for new world monkeys (CMS, Oriental Yeast, Tokyo, Japan) and was given regularly twice a day together with wet mash, supplements, and a favorite food into a cup attached to the cage. Pellets were given every morning (9–10 AM) and another meal was served at around 4 PM. The cage was cleaned by service staff every day before noon.

#### Animals

Ten marmosets were used for this study. Details about each marmoset as well as duration of the experiment (age on the first and last day) are reported in Table 1.

**TABLE 1:** Period of study and age range for each marmoset participating in either *Experiment 1* or *Experiment 2*.

Animal	Entire monitoring period		Experiment 1		Experiment 2	
	Start date (age in days)	End date	Start age (days)	End age (days)	Start age (days)	End age (days)
Sub-adult						
Chomo ♂	2017/8/1 (195)	2018/3/31	204	435		
Lungma ♀	2017/8/1 (195)	2018/3/31	204	435		
Azuma ♀	2017/8/1 (218)	2018/3/31	218	449		
mean (SD)			208.67 (8.08)	439.67 (8.08)		
Young-adult						
Matter ♂	2017/1/23 (167)	2018/3/31	368	599	187	480
Horn ♂	2017/1/23 (167)	2018/3/31	368	599	187	480
Turumi ♂	2016/11/20 (163)	2018/3/31	428	659	184	477
Kano ♀	2016/11/20 (163)	2018/3/31	428	659	184	477
mean (SD)			398.00 (34.64)	629.00 (34.64)	185.50 (1.73)	478.50 (1.73)
Symptomatic transgenic						
Kirishima ♂	2016/9/18 (185)	2017/7/8			185	478
Asymptomatic transgenic						
Ibuki ♂	2016/9/18 (185)	2018/3/31			185	478
Zao ♂	2016/12/19 (170)	2018/3/31			183	476
mean (SD)					184.00 (1.41)	477.00 (1.41)

Seven marmosets housed in the same room were recruited from the breeding colony to measure baseline activity and compare daily activity in different age groups (Experiment 1). To facilitate their habituation, they were moved to the single housing environment at least a week before starting the measurements. We divided the monkeys in two groups according to their age: sub-adults and young-adults (Missler et al., 1992). Three marmosets (one male and two females, aged 208 ± 8 days on the first day of daily activity measurements) were assigned to the sub-adult group; whereas another four marmosets (three males and one female, aged 398 ± 34 days) were assigned to the young-adult group.

For Experiment 2, three marmosets (three males, aged 184 ± 1.4 days) were recruited from the polyglutamine disease model transgenic marmoset line (second generation) recently established in our laboratory (Tomioka et al., 2017). Polyglutamine diseases are heritable neurodegenerative disorders caused by expansion of trinucleotide repeats for the polyglutamine tract (Nóbrega and De Almeida, 2012). This animal model typically shows a variety of motor disease symptoms characterized by progressively lower daily activity (Tomioka et al., 2017). To compile an age-matched wild-type control group, we used data from the young-adult group.

#### Period of Daily Activity Measurement

Information about the duration of each experiment and the animals enrolled is provided in Table 1. In both experiments, animals were moved from the large cage for family housing to the individual cage (30 cm W × 60 cm H × 50 cm D) located in the same breeding room.

### Equipment

**FIGURE 1:**
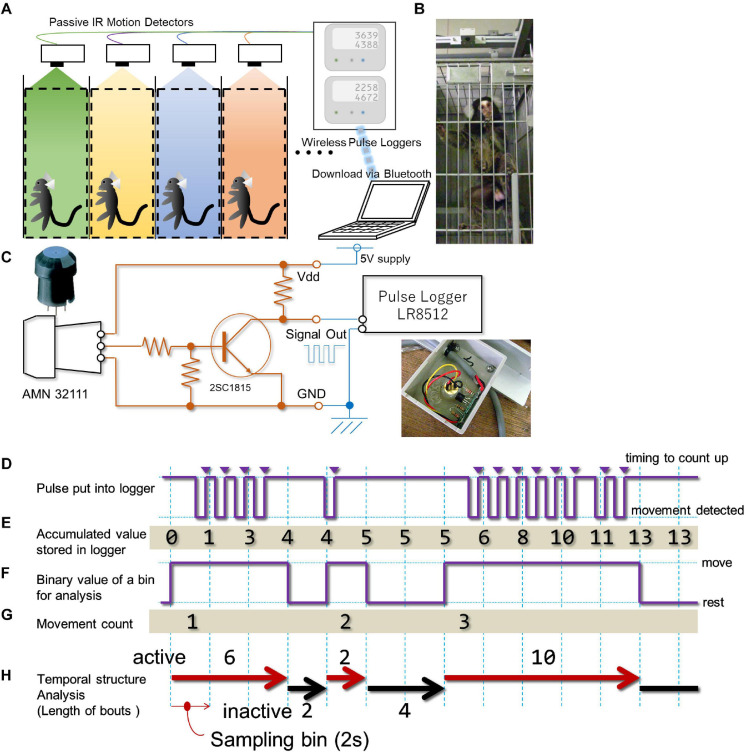
In-cage continuous motility monitoring system for marmosets. **(A)** A schematic diagram of the in-cage continuous motility monitoring system for marmosets. A passive infra-red pyroelectric motion sensor was attached above the ceiling of each cage. Signal from four animals were concurrently and continuously stored by two data loggers, which was downloaded once a week by a PC via blue tooth interface. **(B)** Photo image of the motion sensor (top) and marmoset. **(C)** A circuit diagram of the custom-made buffer amplifier inserted between each motion sensor and data logger. Inset, a photo image of actual installation of the circuit board. **(D)** The sensor sends a negative logic pulse when a movement is detected. This example shows movements of a single marmoset detected during a 28-s period by the sensor. **(E)** The logger counts pulses at each occasion when the logic signal level reaches a high voltage level (triangles) and adds the counts in an incrementally accumulating manner at every 2-s intervals (sampling bin). For the period presented in this example, 13 pulses were detected and accumulated. **(F-H)** Offline processing of the accumulated value from the data logger. **(F)** We converted the accumulated value retrieved from the data logger into a series of binary queue so that it could represent whether or not the increase in the accumulated value occurred every 2 s. **(G)** With our definition of movement, three movements were detected. **(H)** From the binary queue of movement **(F)**, we computed indexes for the successiveness of each phase (active and inactive phases, length of movement bout, length of the inactive phase). See methodology for details.

A commercially available passive infrared pyroelectric motion sensor (AMN32111; Panasonic, Osaka, Japan) was used to detect the movement of each marmoset within their cage (Figures 1A,B). Although this sensor was originally designed to detect subtle movements made by humans from more than two meters above, it can also detect the movements of small animals such as rodents (Brown et al., 2016). We were able to confirm that it can also detect the movements of the common marmoset with a precision of several centimeter (Supplementary Document 1 and Supplementary Videos 1, 2). To send TTL-level count pulses to a data-logger upon occurrence of every movement, we attached a transistor buffer circuit (Figure 1C) housed inside a small plastic box (35 × 53 × 11 mm) together with the sensor (Figures 1B,C). Then, the TTL pulse from the circuit was wired to a compact-sized pulse logger (LR8512; Hioki, Nagano, Japan). This type of data-logger can count the number of TTL pulses within a pre-fixed time bin (from 100 ms to 60 min) and store the cumulative number of pulses for each time bin until it reaches the upper limit of sampling bin capacity (500,000 times). We chose 2 s as the minimum resolution for time domain analysis. This allowed us to compile TTL events over 10 days without any interruption. A single data-logger could receive TTL signals from two sensors simultaneously. The data-logger could work without any external controller and function for an indefinite time. Data were transferred approximately once a week to an external personal computer via blue-tooth.

Each sensor was mounted 170 mm above the cage’s ceiling and 170 mm behind the front of the cage (Figure 1B and Supplementary Figure 1). This location of the sensor covers about 96% of the entire space inside of each cage for detecting the animal’s movement (Supplementary Figure 1). A system consisting of 16 sensors and eight data-loggers enabled the monitoring of up to 16 marmosets at the same time. To prevent detection of movements made by marmosets housed in the neighboring cages, we placed stainless-steel separation-boards at both sides of each cage. Each cage row was separated by a passage (2600 mm width), thus any movement of marmosets housed in the cages on the other side was entirely out of detection range of the sensor. Since the sensor’s range of detection is larger than the cage space, human motion (e.g., feeding or cleaning of the cage) was also detected, and it was not easy to dissociate these movements from those made by the marmoset. The estimated period for each operation is up to two minutes for each animal (corresponding to less than fifty to thirty counts (see next section) per day). Marmosets were kept under continuous 24-h monitoring throughout the assessment period.

### Analysis

#### Dataset

Data retrieved from each data-logger were converted to csv format and were subjected to statistical analysis in R^1^. Although this system allowed for continuous uninterrupted recording during the entire experimental period, some animals had to be occasionally taken from their cage for a routine health examination by a veterinarian. Therefore, we manually excluded daily recorded data if such break exceeded 30 min.

#### Data Processing for Analysis

Data sets were composed of a series of values representing the accumulation of TTL pulse counts (Figure 1D) for each sampling bin (i.e., 2 s) and the number of TTL events per sampling bin was counted (Figure 1E). Due to the intrinsic property of the sensor, we noticed that the single sequence of an animal’s movement could generate multiple TTL pulses. Therefore, each single TTL pulse *per se* might not correspond to a single movement. To deal with this sensor’s property, we had to decrease the precision of time for assessment within the movement count. Specifically, we converted this series to a binary queue encoding whether a single detection of movement occurred in each sampling period (2 s, Figure 1F), and concatenate bins until detecting a state where no movement was detected within a single sampling period. We defined this concatenated bin as the “bout,” and it was used as a basic unit to count the amount of the marmoset’s activity. Subsequently, the following indices were used to parameterize the day-long, binary time-series. a. Gross activity per day (day-long activity) representing the number of movements (Figure 1G) per day (24 h). b. Diurnal activity per day (day-time activity) representing the number of movements during daytime for each day (from 7 AM to 7 PM, light period of the colony room). c. Diurnal index obtained by dividing daytime activity by day-long activity. d. Daily cyclic activity representing the duration of motility in a daily cycle. This index was derived by counting the number of movements per minute (Figure 1G) throughout the experimental period, segmented on a weekly basis (from Sunday to Saturday), and the auto-correlation analysis yields for this weekly time series after applying smoothing (moving average around 9 bins weighted homogeneously). e. Hourly activity representing the number of movements per clock hour throughout the experimental period (represented as weekly average).

#### Intermittent Bursts of Movement (Length of the Movement Bout and Inactive Phase)

While deriving the above indices (Figures 1D,E), we noticed that each movement frequently lasted more than one bin (i.e., more than 2 s, Figure 1F). This intermittency was quantified by computing the length of movement bout and inactive phase. The length of movement bout was quantified by counting the number of back-to-back bins with an active state, followed by unit conversion from bin numbers to seconds (Figure 1H). The length of the inactive phase was quantified by counting the number of bins interposed between two active states, followed by the same unit conversion as for the active state (Figure 1H). To evaluate longitudinal changes with respect to the length of movement bout or inactive phase, the third quartile point of these phases in weekly data was obtained for the entire period.

#### Statistical Analysis

A probability <0.01 was considered significant for all statistical tests unless otherwise mentioned. In Experiment 1, a two-way ANOVA was conducted for effects on individuals and age differences in day-long activities, and the Kruskal-Wallis rank sum test was used for assessing the inter-individual difference in diurnal index. The Friedman test was used to assess the inter-week difference of the length of movement bout and inactive phase. The Wilcoxon rank sum test was used for assessing the difference between two age groups with respect to day-long activity. The paired *t*-test was applied when comparing hourly activity in daytime activity profiles. In Experiment 2, the Wilcoxon rank sum test was used to compare each index before and after the onset of symptoms. The Friedman test was used for assessing the inter-week difference of daytime activity, as well as the length of movement bout and inactive phase. All statistical analyses were carried out using built-in functions in R or the “exactRankTests” package in R.

## Results

### Experiment 1

#### Different Day-Long Activity Between Sub-Adult and Young-Adult Animals

**FIGURE 2:**
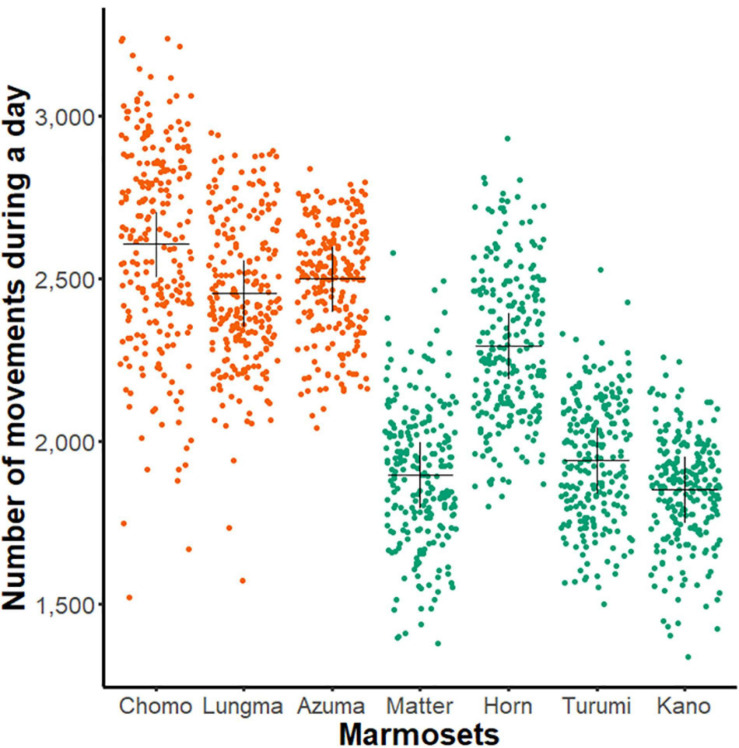
Day-long activity of sub-adult and young-adult marmosets. A comparison of day-long activities (ordinate, aggregated number of movements, see Figure 1G) of each marmoset (abscissa, *n* = 7, name of each marmoset is specified). Each dot represents the net activity for a single day. The black cross indicates mean for all measurement days for each animal; orange, sub-adult group and green, young-adult group.

First, we compared each animal’s amount of day-long activity by plotting the data compiled during the entire recording period separately for all healthy animals (Figure 2).

Using two-way ANOVA, we found a significant main effect in both individuals [*F*(5, 1606) = 126.84, *p* < 0.01] and age-groups [*F*(1,1606) = 2216.8, *p* < 0.01]. This result suggests that the amount of day-long activity changes during development even among young marmosets.

#### Marmosets’ Behavior Is Characterized by a Periodicity of 24 h

**FIGURE 3:**
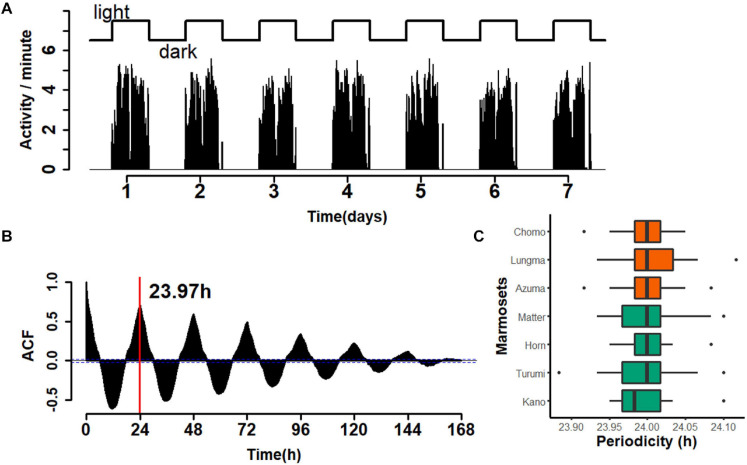
Periodicity of 24 h for marmoset activity. **(A)** An example of a week-long, continuous recording for a single animal (Kano, recorded in week 23). Top, light–dark cycle of the breeding room. Bottom, aggregated number of movements for each minute. Movements were highly restricted during the light period. **(B)** Auto-correlogram of marmoset activity in the same week of the same animal as in panel **(A)**. Red line, estimated duration of a single cycle (23.97 h for the week). **(C)** Median ± inter-quartile range of the cycle duration for each marmoset is shown with the ordinal box plot. The color codes are the same as those used in Figure 2.

Next, we evaluated the periodicity of activity during the circadian cycle. As before, we compared the amount of day-long activity for each animal during the entire recording period. A representative example over a one-week period for a single animal is shown in Figure 3. The activity count was limited during the light period and almost zero during the dark period (Figure 3A). A time series for every week throughout the recording period (re-binned for every minute) was constructed and the auto-correlogram for one week for each monkey was computed (Figure 3B). This autocorrelation analysis yielded an estimate of the duration of a single cycle; in the specific case presented, it amounted to 23.97 h. A comparison for all seven marmosets (Figure 3C) revealed a median daily cycle of 24.001 h for the sub-adult group and 23.996 h for the young-adult group (Wilcoxon rank sum test, *W* = 7132, *p* = 0.1895). These results indicate that marmoset daily life is marked by light cycles in the colony room; however, this periodicity is equal between the two marmoset groups.

#### Pattern of Diurnal Activity and Activity During Daylight

**FIGURE 4:**
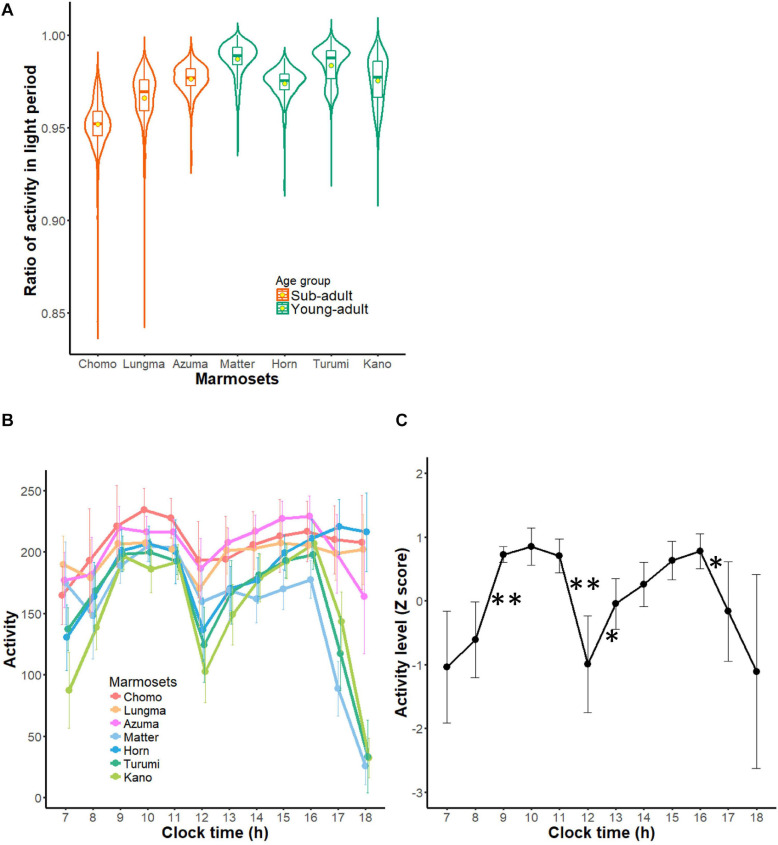
Diurnal activity pattern of common marmosets. **(A)** Diurnal index of each marmoset during all recording periods is represented in the form of a violin plot. Contour indicates the histogram for indexes during all recording periods and the box plot within each contour indicates the medians (horizontal bars) and means (circles). **(B)** Hourly activity of each marmoset during daytime (mean ± SD). Each color represents each marmoset (warm colors for sub-adult groups and cold colors for young adult group are used to match those of other figures). **(C)** A collective profile of daytime activity of marmosets. This plot shows mean ± SD of all seven marmosets from each marmoset’s Z-scored profile. ^∗∗^1% significant difference from the next hour, *5% significant difference from the next hour.

We further characterized the pattern of activity in the active period within each daily cycle (diurnal activity pattern). Although we found some variation in the diurnal index among subjects (from 0.9518 to 0.9870, Kruskal-Wallis rank sum test, Chi-squared = 798.68, *df* = 6, *p* < 0.01), this result indicates that almost all activity was observed during the light period (Figure 4A). Such finding is consistent with the common marmoset being a diurnal animal. Moreover, as the diurnal index was higher in the young-adult group (Wilcoxon rank sum test, *W* = 134440, *p* < 0.01), night time activity was relatively higher in the sub-adult group, suggesting that the diurnal pattern formed during development.

Next, we computed and compared activity per hour during daytime for each marmoset (Figure 4B). Activity was triggered by turning on the light of the housing room and reached its first peak around 9 AM. Then, it decreased until reaching the trough (termed a “siesta” break) around noon. After that, activity increased again, culminating in a second peak around 3–4 PM. Averaging this hourly activity (converted to z score) for each animal (Figure 4C) revealed a remarkable bimodal daily pattern. This bimodality indicates that daytime activity could be divided into two segments: before and after the “siesta” break. Following the second peak around 3–4 PM, four marmosets comprising three young-adults and one sub-adult started exhibiting a decline in activity 2 h before the light went off (paired *t*-test, *t* = 3.0151, *df* = 12, *p* = 0.01076); whereas three marmosets comprising two sub-adults and one young adult kept active even as the light went off (Figure 4B). This result might suggest that the diurnal, bimodal rhythm had not yet developed in the latter three marmosets. Overall, the lower diurnal index in the sub-adult group could indicate a not yet developed day-night cycle; whereas a smaller variance of activity during daytime could reflect an immature intra-daytime cycle in younger animals. These findings suggest that the daily activity cycle of marmosets is shaped progressively during this age interval.

#### Decreased Daily Activity During the Developmental Period

**FIGURE 5:**
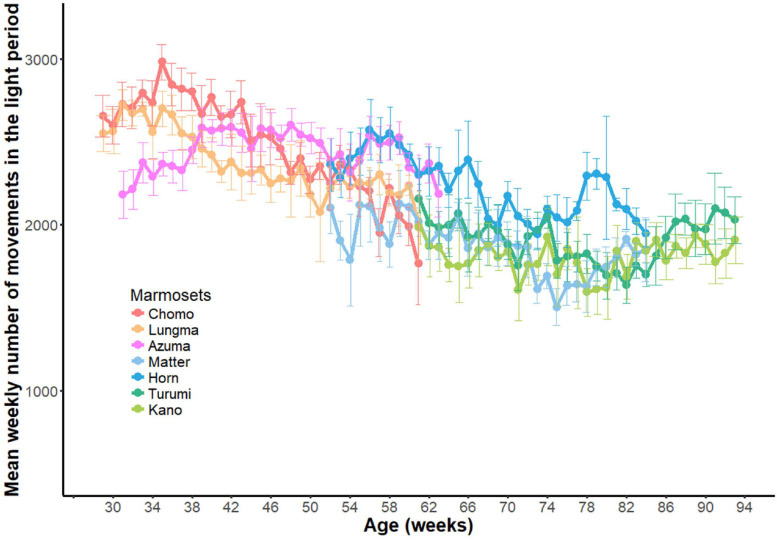
Developmental changes in daytime activity. Longitudinal changes in daytime activities throughout the experiment period (33 weeks) for all seven marmosets. Weekly mean (±SD) for each marmoset was plotted against age.

Longitudinal variations in daytime activity throughout the recording period are shown in Figure 5. Each point indicates the weekly mean (±SD) of daytime activity for each marmoset as a function of the individual’s age. Animals in the sub-adult group exhibited a progressive decrease in activity during a measurement period (reddish color), which was then merged to the level in the young-adult groups (bluish color). By assuming a monotonic decrease in activity as a function of age, we computed a linear regression for both animal groups throughout the recording period. We detected a steeper decrease in the sub-adult group (y = −14.625x + 2679.45, *p* < 0.01, R^2^ = 0.415) than in the young-adult group (y = −6.50x + 2064.99, *p* < 0.01, R^2^ = 0.079), where y is the number of movements and x is the age in weeks.

#### Intermittent Nature of Marmoset Activity

**FIGURE 6:**
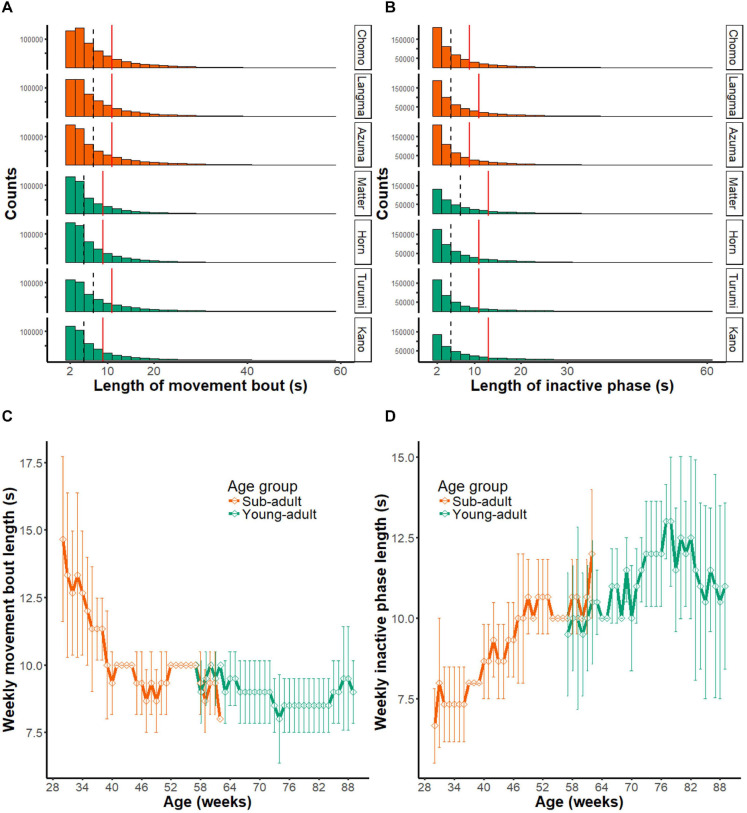
Intermittent nature of marmoset activity. **(A)** Histograms showing the distribution of movement bout lengths during daytime at all periods of monitoring (231 days). The results of seven animals are presented separately. Note that bouts longer than 60 s (0.2% of all data) are not included in the histogram. Dashed line, median of all movement bouts; red solid line; third quantile point of all movement bouts. **(B)** Histograms showing the length of inactive phase during daytime at all periods of monitoring (231 days). Note that inactive phases longer than 60 s (2.6% of all data) are not included in the histogram. Dashed line, median of all movement bouts; red solid line, third quantile point of all movement bouts. **(C)** Longitudinal changes in the length of movement bout. Group mean (±SD) of the index is plotted as a function of mean age of animals. Note that the movement bout decreased as a function of age in the sub-adult group, but the young adult group did not show a significant change. **(D)** Length of the inactive phase is shown in the same format as in panel **(C)**. Note that the length of inactive phase continuously extended throughout the observed age range.

Figure 6A shows the length of movement bouts during the entire recording period in each marmoset. As indicated by the median score (interrupted vertical line), each activity bout lasted several seconds. The third quartile point (red line) indicates a trend associated with long-lasting movements. By tracing the scores for the entire assessment period (Figure 6C), we found that the movement bout decreased as a function of age in the sub-adult group (Chi-squared = 62.873, *df* = 32, *p* < 0.01) but not in the young-adult group (Chi-squared = 44.936, *df* = 32, *p* = 0.6421) by the Friedman test. These results suggest that the length of each movement bout decreases during the early marmoset developmental period.

We made a similar comparison for the inactive phase (Figures 6B,D). In contrast to the movement bout, the length of the inactive phase became progressively longer within the observed age range. Based on the Friedman test, this longitudinal change occurred in the sub-adult group (Chi-squared = 74.708, *df* = 32, *p* < 0.01), as well as in the young-adult group (Chi-squared = 53.732, *df* = 32, *p* = 0.009451).

Overall, the length of movement bout indicates that marmoset activity is intermittent and consists of a bout of several seconds followed by an inactive period. It also suggests that the decrease in activity during development may be explained by both a shorter activity bout as well as a longer resting period between bouts.

### Experiment 2

#### Changes in Motility in a Disease Marmoset Model During Progression of Motor Symptoms

**FIGURE 7:**
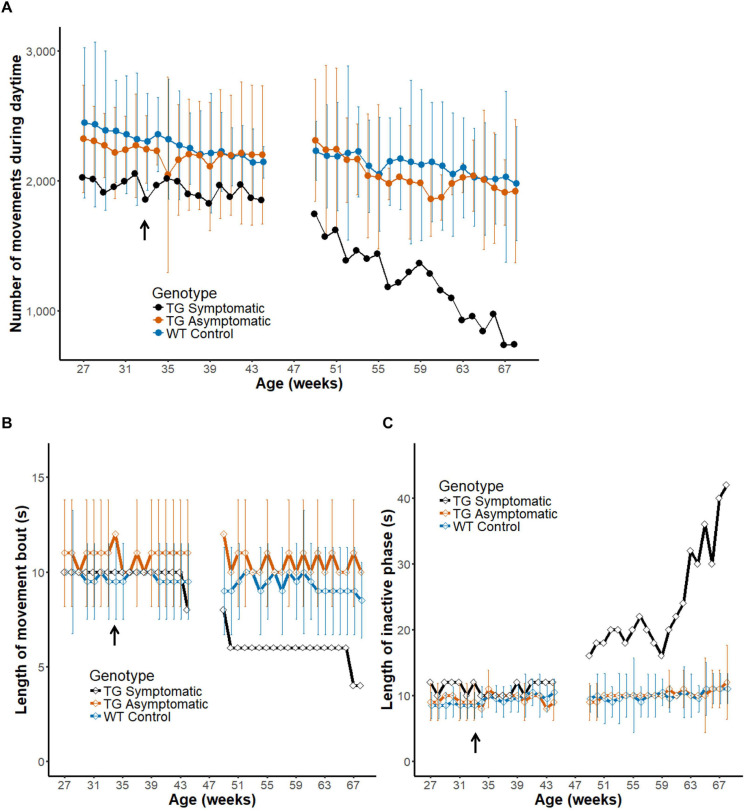
Intermittent nature of activity represents the progression of disease in a transgenic marmoset. **(A)** Number of movements during daytime before and after the onset of diagnosed symptoms (33 weeks of age, arrow in the horizontal axis) in a transgenic marmoset (*n* = 1, black) and age-matched asymptomatic (*n* = 2, orange) and wild-type (*n* = 4, blue) animals (group mean ± 2 SD). Note that the measurement was paused from weeks 45 to 48 as the transgenic animal was temporality transferred to another cage for a neuroimaging experiment. **(B,C)** Longitudinal changes in the length of movement bout **(B)** and inactive phase **(C)**. Group mean (±2 SD) of the index is plotted as a function of mean age. The movement bout decreased and the inactive phase increased as a function of age after disease onset in the symptomatic transgenic animal. Note that the score obtained for a symptomatic marmoset after disease onset was generally beyond the 2 SD range of that obtained for wildtype controls or asymptomatic marmosets.

So far, we analyzed the pattern of in-cage activity in healthy wild-type marmosets (Table 1). Next, we analyzed the development of the symptom of a transgenic marmoset model of ataxia (*n* = 3). A symptomatic animal (Figure 7A, black line) was diagnosed with onset of symptoms around 33 weeks of age (arrow) (Owari et al., 2016). Daily activity differed dramatically between the period preceding the onset of symptoms or their earlier phase (left) and a later phase (right) of progression. Indeed, the motility of the symptomatic animal, as quantified by the daytime activity count, decreased during symptom progression until it dropped to half the initial value. During the latter part of the recording, a steeper monotonic reduction was observed in this symptomatic marmoset, but not in four age-matched wild-type controls (blue line, Chi-squared = 38.314, *df* = 19, *p* = 0.00541) or two pre-symptomatic transgenic marmosets (orange line, Chi-squared = 27.486, *df* = 19, *p* = 0.09384) according to the Friedman test. This result indicates that the method of measuring daily movement developed in this study is sufficiently sensitive to characterize the progressive deterioration of motor symptoms typical of this disease.

#### Alternation of the Intermittent Nature of Daily Activity During Progression of Motor Symptoms

Finally, we evaluated the intermittent nature in daily activity of the symptomatic marmoset during progression of motor symptom (Figures 7B,C) in a similar way as during development (Figure 6). We found a shorter movement bout in the symptomatic transgenic marmoset during the latter weeks, but this decrement was not obtained in both age-matched asymptomatic transgenic marmosets (orange line, Chi-squared = 18.732, *df* = 19, *p* = 0.4741) or age-matched wild-type controls (blue line, Chi-squared = 24.858, *df* = 19, *p* = 0.1653) by the Friedman test. As this measurement may be influenced by the difficulty of maintaining movement for a given period, the changes could reflect the weakening of motor ability caused by disease progression. In addition, we found a gradual lengthening of the inactive phase only in the symptomatic animal (Figure 7C). The increased length of the inactive phase may represent the transient immobilization of movement caused by ataxia and observed also in human patients. Furthermore, this result suggests that measuring intermittent movements could serve as a convenient biomarker of disease progression in the ataxia model, as it could be evaluated based on a shorter assessment period of daily activity.

## Discussion

We developed an in-cage motility monitoring method for marmosets using a passive infrared motion detector, and examined its feasibility during long-term operation (Figure 1). Monitoring of seven animals was successful and without any interruption over 231 days. Activity profiles, including gross daily activity and daytime hourly activity, could be extracted from the data obtained for each marmoset. Furthermore, this method successfully assessed the slow, long-term changes in motility occurring in a marmoset model of human disease. We conclude that the in-cage motility monitoring system is suitable for the longitudinal assessment of marmoset motility.

In the past, various methods for measuring animal motility have been devised. One approach is to use a wearable, microfabricated tiny logger device packaged with micro sensors (Mann et al., 2005; Sri Kantha and Suzuki, 2006; Hoffmann et al., 2012; Melo et al., 2016), whereby changes in acceleration are detected and accumulated over several seconds. The main limitations are the capacity of the data logger and a practical resolution < 0.1 Hz. A popular alternative is to place the sensor in the space surrounding the animals, rather than on the animal itself. Video tracking is commonly used in assays such as the open field test, whereby the travel distance is derived from the locomotion trajectory (Noldus et al., 2001). Recent technological advances have made video tracking devices sufficiently small for their use as an in-cage monitoring system for rodents (Singh et al., 2019). Further, by using multiple cameras or with an additional depth sensor, it is possible to track an animal’s whereabouts in the three dimensional space, including in primates (Ballesta et al., 2014). The disadvantage of such a system is its rather large, fragile, and complex structure when applied in the cage (Yabumoto et al., 2019). Unless it is built a new, it is difficult to make major modifications to an existing animal husbandry facility as it may affect an animal’s physical and mental status. In this study, we overcame this problem by employing a passive movement detector.

### Selection of the Motion Detection Mechanism

Currently, an animal’s movement is detected by physically interrupting the photo-beam, or by recording changes in thermal distribution in space as a function of time. The “photo-beam” method senses the interruption of a near-infrared light beam upon the animal’s passage. However, the area of detection is limited by the width of the photo beam (typically < 10 mm) and, therefore, numerous sensors are required to cover a large three dimensional space when detecting marmoset movement (Pearce et al., 1995). In contrast, the second method locates the animal based on a particular infrared wavelength emitted by the organism. Accordingly, the signals sent back at every change in position are used to track the animal’s movement. Importantly, one sensor can cover a wide three dimensional space, meaning that a single sensor is sufficient to cover almost the entire area of a typical marmoset cage without any modification to the cage itself. This type of sensor is used to evaluate rodents’ activity within a single enclosure (Masuo et al., 1997) and has been recently applied to measure the activity of single animals housed simultaneously in different cages (Brown et al., 2016; Matikainen-Ankney et al., 2019). Based on these characteristics, we chose this method to detect marmoset movement in their home cage.

### Long-Term Recording

A continuous series of data points obtained by in-cage measurements can potentially unmask the periodicity hidden in animal behavior. The profiles obtained from all marmosets in this study showed a cyclic, 24-h transition in activity level, with almost all activity occurring during the light period. These findings match the diurnal nature of marmosets (Menezes et al., 1993; Hoffmann et al., 2012) and are in line with studies on their circadian rhythm (Erkert, 1989).

The bimodal profile of daily activity is consistent with the reported profiles of two prominent active phases during daytime (Erkert, 1989; Castro et al., 2003; Melo et al., 2013). Although peak level before noon may be affected here by the husbandry routine, lower activity around noon may reflect the marmoset’s specific behavioral pattern previously observed in free-ranging individuals (Castro et al., 2003). The hourly variation in daily activity observed here implies that any comparison in motility among or within subjects might be validated only if data are recorded at a comparable time of the day. In this sense, in-cage 24-h measurements provide an excellent source of information.

The marmoset’s moderately long lifespan is advantageous when investigating primate aging or slowly progressive diseases (Maclean et al., 2000; Tardif et al., 2011). Recently, alternations in the daily rhythm have been associated with neurodegenerative disease (Leng et al., 2019). An altered synchronization to light has been observed in aged marmosets (Gonçalves et al., 2016) and REM sleep behavioral disorder has been reported in a Parkinson’s disease marmoset model (Verhave et al., 2011). Considering the monophasic pattern of sleep (Hoffmann et al., 2012), marmoset is an ideal species for studying sleep disorder. Our system for long-term continuous recording could evaluate changes characterized by such relatively slow time constant.

We found that marmoset activity differed among two age groups. Various stages are proposed as developmental milestones in marmosets (Missler et al., 1992; De Castro Leao et al., 2009; Schultz-Darken et al., 2016). The animals used in the present study corresponded to the sub-adult and young-adult developmental phases (Missler et al., 1992). Lower overall activity in the relatively more mature young-adult group suggests that the motility measurement obtained with this system reflects the profile of each developmental stage. Melo et al. (2016) reported higher daily activity in juvenile marmosets compared to their parents. A gradual decline in activity from sub-adults to young-adults may be a function of development. Even though early maturity of marmoset behavior has been reported (Wang et al., 2014), the time-course of subsequent motor development is less known. Hence, the proposed movement monitoring system may help gather life-long data essential when using marmoset as the animal model of human disease at different developmental stages.

### Measurement From a Transgenic Marmoset Model of Human Ataxia

Our system was capable of quantifying the monotonic decline in motility of transgenic marmosets mimicking human polyglutamine disorder (Tomioka et al., 2017) during the entire course of disease progression (Owari et al., 2016). In a previous study, using a rodent model of human neurodegenerative disorder, the onset and time-course of disease progression were identified by measuring animal motility (Norflus et al., 1998; Rudenko et al., 2009; Holter et al., 2013). Our study upgraded those observations in two important ways. First, we applied the method to the marmoset monkey, which is a closer relative to humans. Second, we covered the entire life span of each transgenic marmoset and successfully characterized the pre- and post-symptomatic state.

It should be noted that scattered sampling of motility has already been reported in a pharmacologically induced marmoset model (Pearce et al., 1995; Ando et al., 2008; Verhave et al., 2011; Phillips et al., 2017). However, this is the first such study on genetically induced animals. Using linear regression, we successfully estimated the quantitative time constant of disease progression at -46.74 per week during the last 20 weeks of measurement (Figure 7A). The ability to accurately quantify the decline in motility is crucial for establishing new intervention strategies and assessing the effectiveness of therapeutic agents.

### Temporal Structure Analysis

Animal motility can be characterized by analyzing its temporal structure (De Visser et al., 2006; Umemori et al., 2009; Blum et al., 2014). Aside from the circadian rhythm, an animal’s behavior can exhibit cycles of activity and inactivity, as well as include a shorter “ultradian” rhythm (Honma and Hiroshige, 1978; Blum et al., 2014) or sub-hour and sub-minutes cycles. The circadian rhythm and the shorter cycle could be controlled by distinct brain mechanisms (Blum et al., 2014) and deteriorate independently under specific brain disorders (Nakamura et al., 2007). Intermittency of movement, i.e., the short-cycle rhythm, has been successfully used to characterize motility profiles among different rodent strains (De Visser et al., 2006), as well as symptom progression in the transgenic motor disease model (Hickey et al., 2005). Using a comparable time resolution, Goulding et al. (2008) found a specific “temporal structure” within a short active cluster for intake behavior, which differed in mutant obesity mice.

Time resolution of our system enabled us to characterize short-cycle movement in the marmoset home cage (Figure 1). Indeed, we found that the duration of both active and inactive phases varied with animal age and there was a steep increase in the length of the inactive phase after symptom progression in a transgenic marmoset. From these results, we conclude that short-cycle movement could serve as a marker to characterize the motility of both wild-type and transgenic model marmosets. Moreover, it could be applied to evaluate the extent of ataxia as a function of disease progression in other model animals (Hickey et al., 2005) or to characterize the motility of non-human primates using temporal structure analysis (Goulding et al., 2008).

### Simultaneous Measurement From Various Marmosets in the Same Colony

The most important advantage of our system is its simplicity. It is made exclusively by off-the-shelf electrical parts and can be easily installed on the marmoset’s home cage, making it possible to simultaneously monitor the motility of numerous animals at low cost. The measurement of large numbers of animals has a clear advantage for non-human primates. For example, their intricate, albeit highly evolved, structure of the musculoskeletal and neural systems renders their behavioral pattern highly complex. For this reason, quantitative evaluation of behavior has been challenging, as well as time- and energy-consuming due to a one-by-one approach. Sample size is becoming crucial for contemporary studies of non-human primates (Prescott, 2010).

Another advantage arising from simultaneous behavioral recording of many animals is the possibility to extract “common-mode noise” originating from a shared housing environment, and purify the recorded measurement by eliminating such noise. Generally speaking, animal’s behavior is driven either by the internal state of the body or by stimuli arising from the external environment A classical, session-based measurement (Wahlsten, 2001) aims to dissociate the latter from the former. To assess auditory response, for example, the experimenter presents the same auditory stimulus repetitively in a sound-proof chamber and extracts the ensuing common neural response (Bendor and Wang, 2007). The disadvantage of behavioral measurement in the home cage is represented by the difficulty to control environmental factors. In a normal marmoset colony, a number of auditory stimuli (e.g., vocal calls, movement of other animals or human staff providing daily care) may occur at times that are fairly unpredictable by the experimenter. Therefore, repetitive presentation of the same sound may generate a type of behavior that was actually triggered by other external events occurring coincidentally with the experimental sound. As a consequence, results manifest lower reproducibility between trials or individuals. One way to overcome this limitation is to monitor stimuli-driven behaviors concurrently in all animals. Known as the yoked control design, this method records the behavior triggered by the same controlled and uncontrolled environmental stimuli in all animals. The experimenter then obtains purified data by subtracting the component associated to the same environmental signal from all subjects.

Finally, the proposed method has another advantage in terms of reproducibility. Some marmosets are difficult to habituate to the experimental set-up, and the way and extent of habituation may differ between individuals. Measurement in our method is performed in the most familiar space for the animals and cancels out the irrelevant variance generated by acquiring data from a large number of subjects.

### Limitations

Lastly, it is worth mentioning the four potential limitations of the proposed activity sensing system. Firstly, our system cannot assess the activity of individual marmoset when they are housed with other animals. This is because the sensor system we used cannot track a specific heat source. There are other systems that are dedicated to this objective (Mann et al., 2005). Secondly, the sensor used in this study was originally designed for the precise detection of human movements, but not for small and versatile animals like a common marmoset. Consequently, it is not assured that the system can detect a variety of movements without failure even when animals were within the field of detection of sensor. Third, due to the intrinsic properties of the sensor, the sensitivity of detection changes as a function of distance between the sensor and animals (Supplementary Figures 2C,D). Therefore, movement detection could be biased toward a situation when animals stayed in the upper part of the cage. Fourth, the system cannot dissociate different types of movement (e.g., locomotion or movement of body parts, Supplementary Videos 1, 2) because the sensor simply detects the variance of temperature within the field. The pose estimation-based motion analysis approach (Mathis et al., 2018; Mathis and Mathis, 2019), for example, is superior for this objective. Overall, a potential user of this system needs to be aware that our system can provide a relative, but not absolute, evaluation of an animal’s motility. While it is useful to compare the motility among different animals or that of the same animal on a different day, it may not be straightforward to compare with the motility evaluated by other systems.

## Data Availability Statement

The original contributions presented in the study are included in the article/Supplementary Material, further inquiries can be directed to the corresponding author.

## Ethics Statement

The animal study was reviewed and approved by the committee on the ethical issue in animal experiments of the National Center of Neurology and Psychiatry.

## Author Contributions

MK and KS designed, manufactured, installed the in-cage motility measurement system, and wrote a draft of the manuscript. MK and AK performed the measurements. MK analyzed the data. TN, NN, KO, and KS generated the transgenic animal used in the study. All authors contributed to the final version of this manuscript.

## Conflict of Interest

The authors declare that the research was conducted in the absence of any commercial or financial relationships that could be construed as a potential conflict of interest.

## Footnotes

1 https://www.r-project.org/
