# Supplementary material for: Motility Profile of Captive-Bred Marmosets Revealed by a Long-Term In-Cage Monitoring System
Source: Front Syst Neurosci. 2021 Apr 15;15:645308. doi: 10.3389/fnsys.2021.645308 (PMC8081884; doi:10.3389/fnsys.2021.645308)
Supplement: Supplementary file 1 [file Presentation_1.pdf]

Supplementary Material

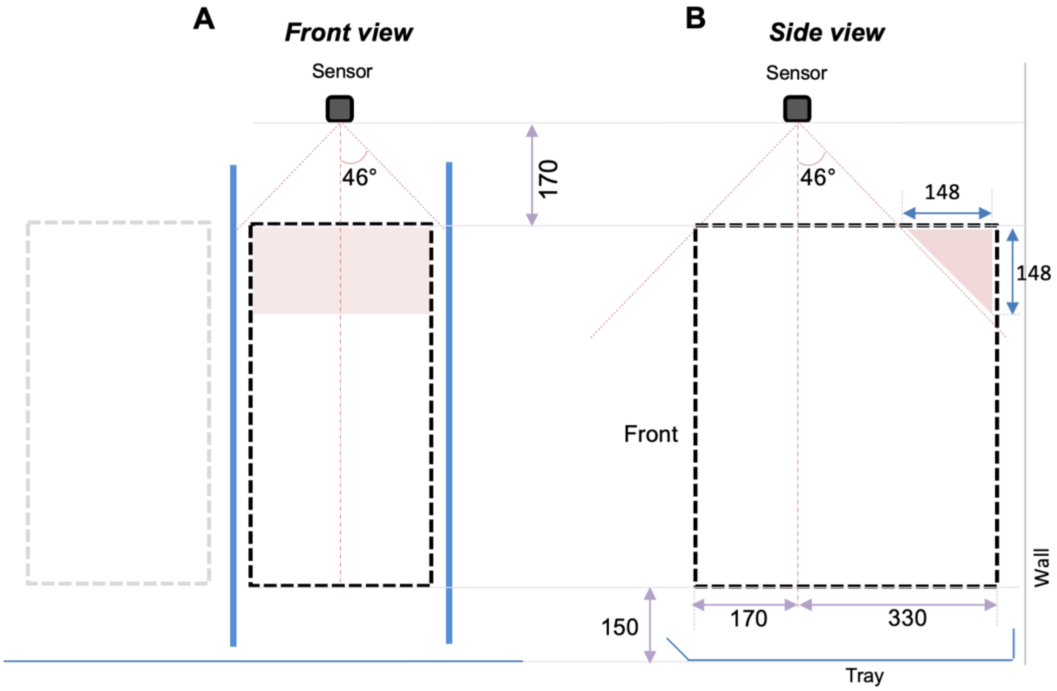


**Supplementary Figure 1. Installation of the detector in the marmoset cage.**

Construction of both the existing housing room and marmoset cage forced us to install each detector 170 millimeters above the cage's ceiling. This position could also detect the movements of animals in neighboring cages (A). We therefore installed stainless-steel-made separation boards between each cage (blue line in A). Since the position of the sensors is slightly lower to cover the entire cage, we could not avoid having a blind spot (red shade) in the cage where the detection of movement was not possible. To minimize this blind spot's effect on the total activity counts, we installed the detector at a location 80 mm toward the front of the cage (B) from the center position, since most animals prefer climbing up and down in the front but not back of their cage. As a result, this installation resulted in a blind spot in the rear corner just under the cage's ceiling (red shades) that occupies only 4% of the entire space in our cage.

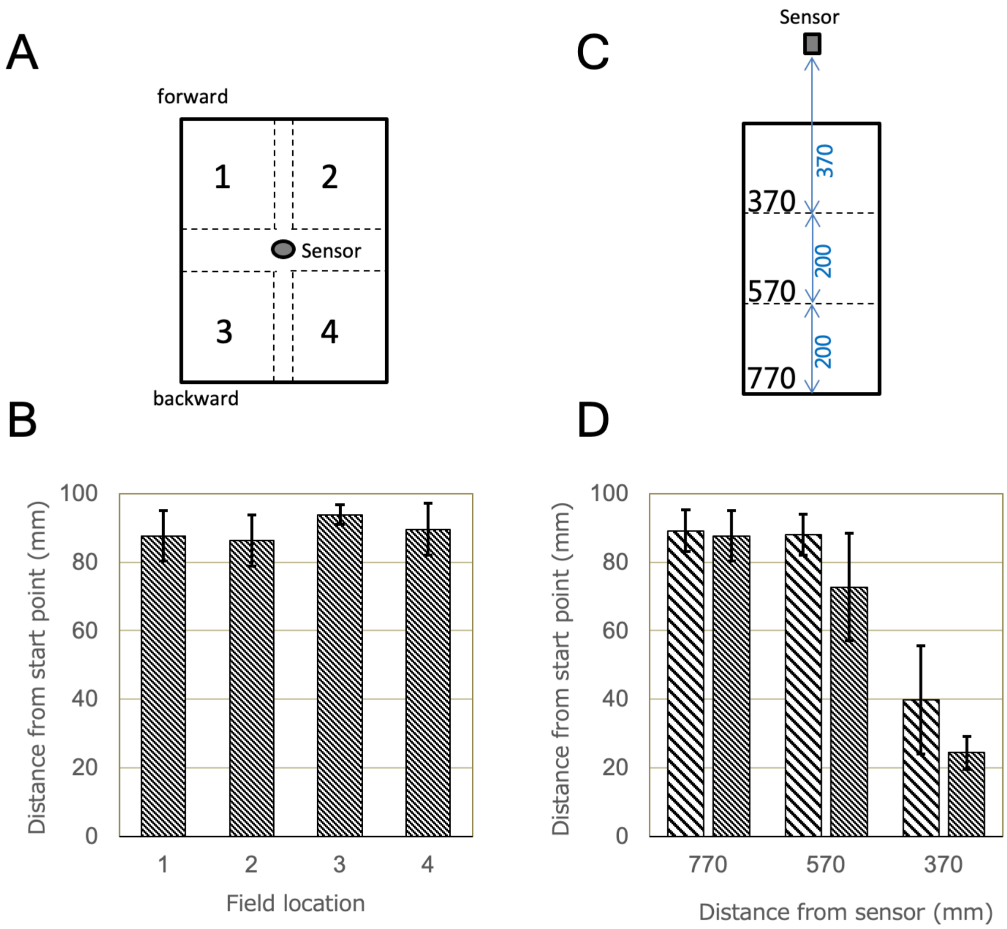


**Supplementary Figure 2.** Detection resolution of marmoset movements**.**

The marmoset was kept in a transfer box (wooden with mesh top) for a short period, and an experimenter moved the box repeatedly with constant speed manually under the sensor by pulling the trolley handle that was attached to the box. The position of the box was continuously measured by a laser beam displacement sensor (CD22-100VM122, Optec FA, Kyoto, Japan). The initial position of box was placed within the center of each measurement field (one of four field in A) and the moved distance of the sensor was determined by reading the signal from displacement sensor at the timing when the TTL pulse occurred. A, B; Difference in detection precision among the four measurement fields. (A) The rectangular area, corresponding to the cage's floor, has been divided into four fields (500x300mm) (A). (B) Results were obtained by using one marmoset (Monkey B). The plot shows the mean detection point calculated from 20 trials at each location. The spatial resolution of this system is about 90 millimeters, according to this test. The same detection resolution level was confirmed in each field (i.e., field 1-4 in A: $F(1,70)=2.467$, $p=0.121$). C, D; Influence of distance between sensor and animals (C) on the detecting precision. In this test, the same procedure has been repeated in three different heights of the sensor (370, 570, and 770 mm from the floor) and comparison of the detection precision in two marmosets (diagonal and doted shade, respectively). Both marmosets showed significant effects for height (Monkey 1: $F(1,54)= 96.63$, $p<0.01$; Monkey

2: $F(1,54) = 259.9$ $p<0.01$ ) (D). Monkey 1 was tested in field location 4 (A) and Monkey 2 in location 1.

**Supplementary video 1. Arena recording (top view).**

Video recordings (90 sec) were made in the arena enclosed with a transparent plastic box (W300xD500xH600). The marmoset (age:1730 days, BW:434g) was left in the box for the video recording only. Sensors were localized in the center of the arena (770mm from the floor). A comparable detection system described in this paper was used. Beep sounds correspond to the timing of the TTL pulse of the sensor. This video clip shows that the sensor could detect postural changes, large head movements, loitering, and jumps from the floor. Top view.

"Video1.mp4"

**Supplementary video 2. Arena recording (front view).**

This video clip was recorded simultaneously with the supplementary video 1 from top of arena.

"Video2.mp4"
